# The diagnostic value of machine learning for the classification of malignant bone tumor: a systematic evaluation and meta-analysis

**DOI:** 10.3389/fonc.2023.1207175

**Published:** 2023-09-07

**Authors:** Yue Li, Bo Dong, Puwei Yuan

**Affiliations:** Department of Orthopedics, Xi’an Honghui Hospital, Xi’an Jiaotong University, Xi’an Shaanxi, China

**Keywords:** machine learning, meta-analysis, malignant bone tumor, diagnosis, systematic evaluation

## Abstract

**Background:**

Malignant bone tumors are a type of cancer with varying malignancy and prognosis. Accurate diagnosis and classification are crucial for treatment and prognosis assessment. Machine learning has been introduced for early differential diagnosis of malignant bone tumors, but its performance is controversial. This systematic review and meta-analysis aims to explore the diagnostic value of machine learning for malignant bone tumors.

**Methods:**

PubMed, Embase, Cochrane Library, and Web of Science were searched for literature on machine learning in the differential diagnosis of malignant bone tumors up to October 31, 2022. The risk of bias assessment was conducted using QUADAS-2. A bivariate mixed-effects model was used for meta-analysis, with subgroup analyses by machine learning methods and modeling approaches.

**Results:**

The inclusion comprised 31 publications with 382,371 patients, including 141,315 with malignant bone tumors. Meta-analysis results showed machine learning sensitivity and specificity of 0.87 [95% CI: 0.81,0.91] and 0.91 [95% CI: 0.86,0.94] in the training set, and 0.83 [95% CI: 0.74,0.89] and 0.87 [95% CI: 0.79,0.92] in the validation set. Subgroup analysis revealed MRI-based radiomics was the most common approach, with sensitivity and specificity of 0.85 [95% CI: 0.74,0.91] and 0.87 [95% CI: 0.81,0.91] in the training set, and 0.79 [95% CI: 0.70,0.86] and 0.79 [95% CI: 0.70,0.86] in the validation set. Convolutional neural networks were the most common model type, with sensitivity and specificity of 0.86 [95% CI: 0.72,0.94] and 0.92 [95% CI: 0.82,0.97] in the training set, and 0.87 [95% CI: 0.51,0.98] and 0.87 [95% CI: 0.69,0.96] in the validation set.

**Conclusion:**

Machine learning is mainly applied in radiomics for diagnosing malignant bone tumors, showing desirable diagnostic performance. Machine learning can be an early adjunctive diagnostic method but requires further research and validation to determine its practical efficiency and clinical application prospects.

**Systematic review registration:**

https://www.crd.york.ac.uk/prospero/, identifier CRD42023387057.

## Introduction

Malignant bone tumors are diseases caused by the growth and spread of malignant tumor cells in bone tissue and the destruction of bone structure. There are various bone tumor types, such as osteosarcoma, multiple myeloma, and metastatic bone tumors. Such malignant tumors usually occur in different parts of the bones, such as long bones, flat bones, vertebrae, and pelvic bones (1). The clinical manifestations mainly include bone pain, swelling, tumor, and fracture, which can also be accompanied by other types of bone diseases, like osteomyelitis and osteoporosis (2). The prevention and early diagnosis of malignant bone tumors remain challenging and require comprehensive measures, including raising people’s health awareness and enhancing the development of screening and early diagnosis technologies (3).

Currently, the diagnostic modalities for malignant bone tumors mainly encompass imaging examinations, histological examinations, and laboratory tests (4). Imaging examinations include X-ray, CT, MRI, bone scan, etc., which can provide information about bone morphology, structure, density, and metabolism (5). Histological examinations help determine the tissue type of lesions by tissue biopsy or cytologic examination, consisting of needle biopsy, puncture biopsy, and surgical excision of tissue (6). Laboratory tests mainly include hematological and biochemical tests, which can evaluate tumor markers, bone metabolism markers, and other biochemical indicators (7). Among the above diagnostic methods, histological examination is currently the gold standard for the diagnosis of malignant bone tumors because it can clarify the tissue type of the lesion and thus provide guidance for the selection of treatment protocols. However, these diagnostic approaches also have some limitations. For example, imaging examinations have a low detection rate for early lesions, or even fail to detect certain lesions at an early stage; histological examinations require surgery or biopsy, which can cause some trauma and risk to the patients and may sometimes result in misdiagnosis due to insufficient tissue sampling or wrong histological analysis (8); the sensitivity and specificity of tumor markers in laboratory tests are limited, and other diseases may also present elevated levels of certain tumor markers, so laboratory tests cannot be used as the only criteria for the diagnosis of malignant bone tumors. Therefore, more accurate and non-invasive diagnostic techniques for malignant bone tumors are required.

With the development and application of machine learning technology in recent years, its application in the diagnosis and classification of malignant bone tumors has become increasingly promising (9). Machine learning can identify and classify tumors by automatically discovering the patterns and features hidden in the data after training and learning from a large amount of data (10). Meanwhile, Compared with traditional diagnosis, machine learning can train models with large amounts of data to improve the accuracy and precision of diagnosis and avoid the impact of doctors’ personal experience and subjective judgment on the diagnosis results. Machine learning can automatically analyze medical images, clinical features, and other information to quickly complete a large amount of work, reducing the workload of physicians and improving work efficiency (11–13). K. Zhao et al. (14) constructed three DL models based on sagittal, coronal, and axial MR images, respectively, to predict the malignancy of tumors, which significantly improved the diagnostic accuracy of one oncologist and two orthopedic surgeons. Also, these models improved the diagnostic sensitivity of two oncologists, one radiologist, and three orthopedic surgeons. R. Liu et al. (15) found a 4.3% increase in accuracy, a 0.026 increase in AUC, and a 3.4% increase in sensitivity for all radiologists supported by a three-class classification fusion model. Y. He et al. (16) included data on 1,356 bone tumor patients from pathology databases at 5 institutions. The CNN model had an AUC of 0.894 and 0.877 in cross-validation and external testing, respectively, with accuracy similar to that of subspecialists and superior to that of junior radiologists.However, the diagnostic accuracy of these models is currently controversial, and there is a lack of relevant systematic reviews to provide evidence-based support. Therefore, this systematic review and meta-analysis was conducted to evaluate the accuracy of machine learning models based on different modeling variables in the diagnosis and classification of malignant bone tumors, exploring the prospects and limitations of their application in clinical practice and providing evidence-based references for future diagnostic decisions of malignant bone tumors.

## Materials and methods

This study was conducted according to The Preferred Reporting Items for Systematic Reviews and Meta-Analyses (PRISMA 2020) statement (17).

### Inclusion and exclusion criteria

Inclusion criteria: (1) study subjects were patients with malignant bone tumors; (2) study types were case-control studies, cohort studies, nested case-control studies, and case-cohort studies; (3) a machine learning predictive model was fully constructed; (4) studies without external validation were also included; (5) different machine learning studies published in the same dataset were included; and (6) English literature was included.

Exclusion criteria: (1) Meta, review, guideline, expert opinion, etc.; (2) the study only performed predictive factor analysis and did not construct a complete machine learning model; (3) the literature lacked the following outcome indicators of predictive accuracy of machine learning models (Roc, Concordance Statistic(c-statistic), Concordance Index(c-index), sensitivity, specificity, accuracy, recovery rate, accuracy rate, confusion matrix, diagnostic fourfold table, F1 score, calibration curve); (4) validation of only mature scales; and (5) studies on single-factor diagnostic accuracy.

### Document retrieval

A systematic search was performed on PubMed, Embase, Cochrane Library, and Web of Science as of October 31, 2022 for literature on the application of machine learning to assist physicians in the diagnosis of malignant bone tumors. The retrieval used a combination of subject terms and free-text terms. The detailed retrieval strategy is described in **Supplementary Material 1**.

### Data extraction

The literature obtained from database retrieval was imported into the EndNote 20 software for management. After duplicate publications were excluded, the titles and abstracts were read to exclude literature that did not meet the inclusion criteria. Then the full texts of the remaining studies were read to identify the final included literature. A data extraction spreadsheet was developed to extract basic information and model characteristics from the included studies. The extracted data included: first author, title, year of publication, author’s country, study type, patient source, type of malignant bone tumor, number of malignant bone tumor samples, total sample size, number of malignant bone tumor samples in the training set, total sample size in the training set, generation method of the validation set, overfitting method, number of malignant bone tumor samples in the validation set, total sample size in the validation set, treatment of missing values, feature selection method, model type, and modeling variables. The diagnostic fourfold table was made after calculation. Two investigators independently conducted the above literature screening and data extraction and cross-checked their results after completion. In case of dispute, a third-party investigator was asked to assist in the adjudication to reach a final consensus.

### Risk of bias assessment

The risk of bias assessment for the included studies was performed using the QUADAS-2 scale, which includes both risk of bias and clinical applicability evaluation. The assessment was performed independently by two investigators, and in case of disagreement in the quality evaluation, a third investigator was asked to assist in the final decision. Low risk was considered in an item when the data meet the requirements of the item, high risk when the data did not meet the requirements, and unclear risk when the data was not specified.

### Statistical analysis

Stata17 software and STATA’s midas and mylabels package were applied for statistical analysis. The number of true-positive, false-positive, true-negative, and false-negative cases in each study was listed. A mixed-effects model was employed to calculate the combined sensitivity, combined specificity, positive likelihood ratio (PLR), negative likelihood ratio (NLR), diagnostic odds ratio (DOR), and Summary Receiver Operating Characteristic(SROC) for the included literature. SROC curves were plotted to determine the accuracy of machine learning in diagnosing malignant bone tumors. Area Under the Curve(AUC) = 0.5 suggests no diagnostic value at all; 0.5 < AUC ≤ 0.7 suggests low diagnostic accuracy; 0.7 < AUC ≤ 0.9 suggests average diagnostic accuracy; AUC > 0.9 suggests high diagnostic accuracy (18). Forest plots were drawn using sensitivity and specificity. Heterogeneity was measured by I^2^. An I^2^ value of 40% was considered to have significant heterogeneity. The bivariate modeling approach simulates both sensitivity and specificity after logit transformation to explain the inherent negative correlations between sensitivity and specificity that may arise due to different thresholds for different studies (19). PLRs and NLRs were used to plot nomograms to evaluate their clinical applicability. The prevalence of lesions in the pooled study population was used as prior information, and the post-test probabilities for each type of lesion were deduced based on the pooled PLRs and NLRs. Deek’s funnel plot was applied to analyze whether there is potential publication bias in the included studies. A P > 0.05 suggests that there is no publication bias. Subgroup analyses were conducted by modeling variables and model types. The bivariate mixed-effects model requires the number of included models to be ≥ 4. Therefore, only the ranges of sensitivity, specificity, PLR, NLR, and DOR were listed when the number of models was less than 4 in subgroup analyses.

## Results

### Results of literature screening

**Figure 1:**
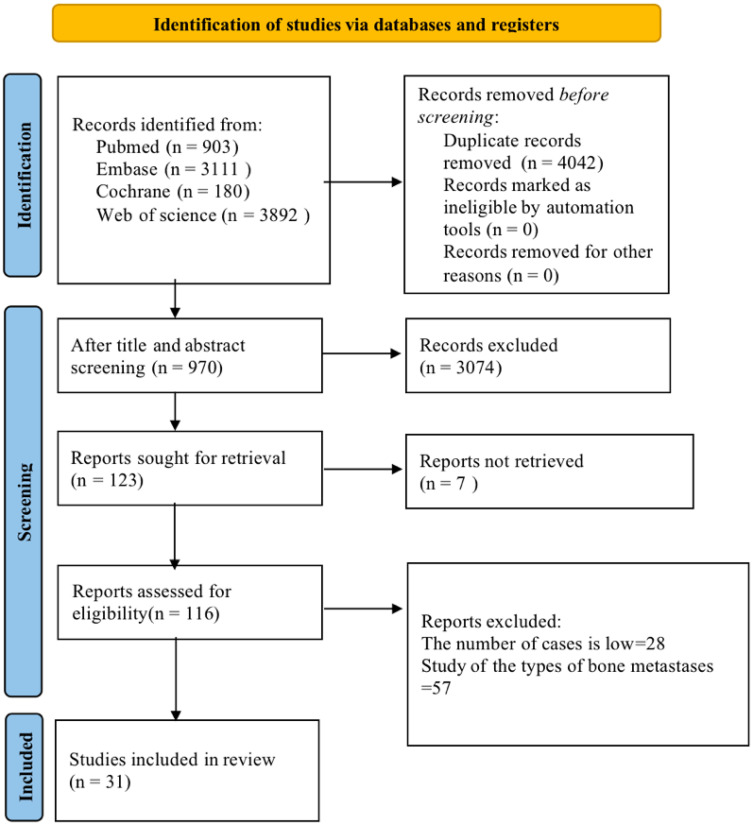
Literature screening flow chart.

Initially, 8,086 articles were retrieved. The retrieved literature was imported into EndNote 20 and then checked for duplication, and 4,042 articles were excluded. The titles of the included 4,044 articles were read to exclude the literature that did not meet the inclusion criteria, such as conference abstracts, animal experiments, etc., and 1,770 articles were left. After the abstract reading, ineligible literature was excluded. Finally, 31 articles were included (14–16, 20–47). The literature screening flow chart is shown in **Figure 1**.

### Basic characteristics of the included literature

The 31 original studies included in our systematic review were published mainly between 2021 and 2022, covering 382,371 samples, of which 141,315 were malignant bone tumor samples. The countries of publication contained China (15, 16, 29, 30, 34, 35, 38, 43, 45, 48), the USA (23), Korea (33, 49), Germany (39, 40), Italy (22, 26, 27), Japan (44), India (28, 37), Spain (32), Thailand (31), and Saudi Arabia (24). The type of study was mainly retrospective. There were 8 multicenter studies (16, 23, 25, 27, 34, 39, 40, 42) and 4 studies from registered database sources (24, 32, 33, 46). Disease studies included pan-studies on malignant bone tumors and studies on different specific malignancies, among which 16 articles were on malignant bone tumors (including 2 articles on malignant bone tumors of the spine only), 6 articles on multiple myeloma, 5 articles on osteosarcoma, and 4 articles on chondrosarcoma. The mainstream splitting method of the validation set is K-fold cross-validation (5-fold or 10-fold). The included studies were mainly internally validated, whereas some were externally validated. The basic information of the included studies is available in **Supplementary Material 2**.

### Model characteristics

Thirty-four models were extracted from the 31 articles. Duplicated models, including radiomics models and radiomics+clinical characteristics models, were constructed by J. Pan et al. (35), R. Liu et al. (15), F. R. Eweje et al. (23), and C. E. von Schacky et al. (40). Among them, additional models based on clinical features were constructed separately by C. E. von Schacky et al. There were 7 types of models: 13 Convolutional Neural Networks(CNN) models, 4 Artificial Neural Networks(ANN) models, 4 Random Forest(RF) models, 5 Support Vector Machines(SVM) models, 4 Logistic Regression(LR) models, 2 Decision Trees(DT) models, and 2 eXtreme Gradient Boosting(XGboost) models. Seven types of modeling variables were covered: 5 CT-based models, 14 MRI-based models, 7 X-ray-based models, 4 pathological image-based models, 3 clinical features-based models, 1 Laser-Induced Breakdown Spectroscopy(LIBS)-based model, and 1 Positron Emission Tomography/Computed Tomography(PET/CT)-based model. LIBS modeling was reported by X. Chen et al. (21), who combined serum-based LIBS with machine learning methods to construct a model using data from 130 patients with multiple myeloma. PET/CT modeling was reported by R. Xu et al. (44), who adopted machine learning methods to improve the differential diagnosis of (24) f-FDG PET/CT images for malignant bone tumors. The model characteristics are available in **Supplementary Material 3**. The diagnostic fourfold table is provided in **Supplementary Material 4**.

### Risk of bias assessment results

The quality of the included studies was evaluated using the QUADAS-2 scale. Most studies enrolled consecutive or randomized cases and avoided case-control designs, with reasonable exclusions. Six papers (16, 20, 23, 38, 40, 47) involved selective inclusion of cases, three papers (20, 21, 38) were case-control studies, and two papers (28, 43) did not specify the type of study, which could lead to potential case selection bias. Three papers (25, 32, 43) were unable to derive a diagnostic fourfold table due to missing data. The risk of bias and clinical applicability evaluation for the rest of the literature were considered low risk. The risk of bias evaluation results are shown in **Supplementary Material 5**.

### Results of meta-analysis

**Figure 2:**
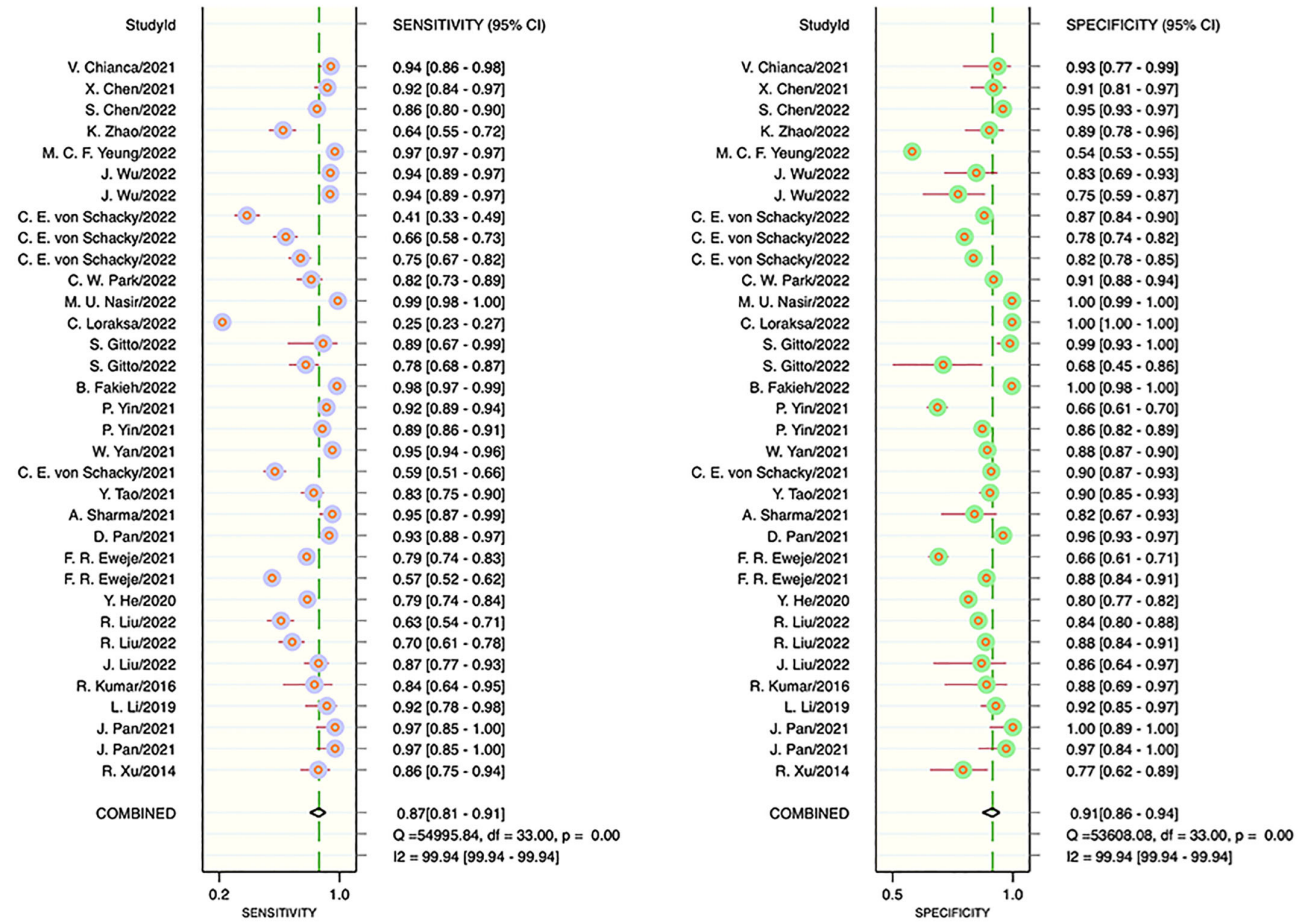
Forest plot for sensitivity and specificity in the training set.

**Figure 3:**
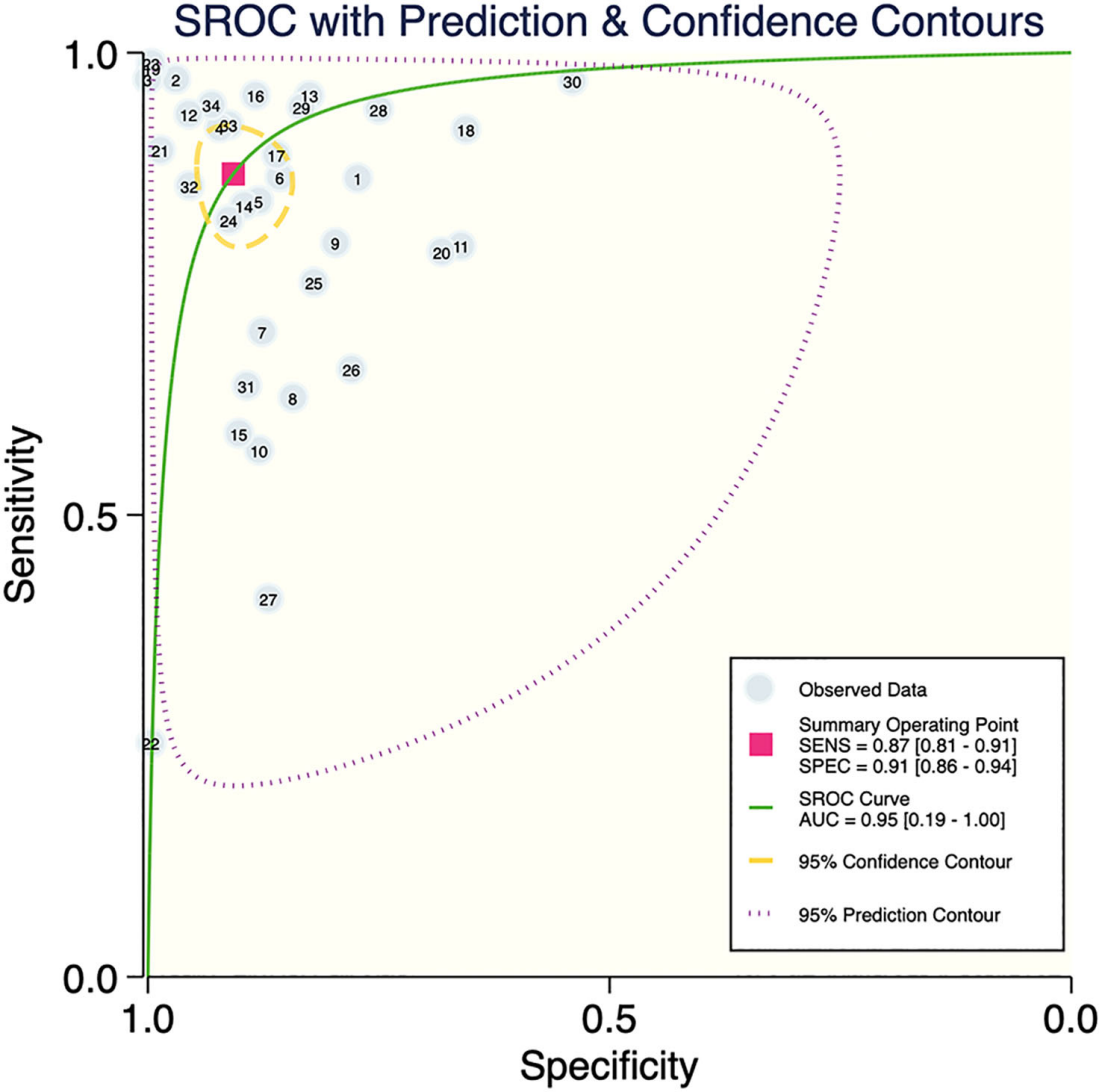
SROC plot of the training set.

**Figure 4:**
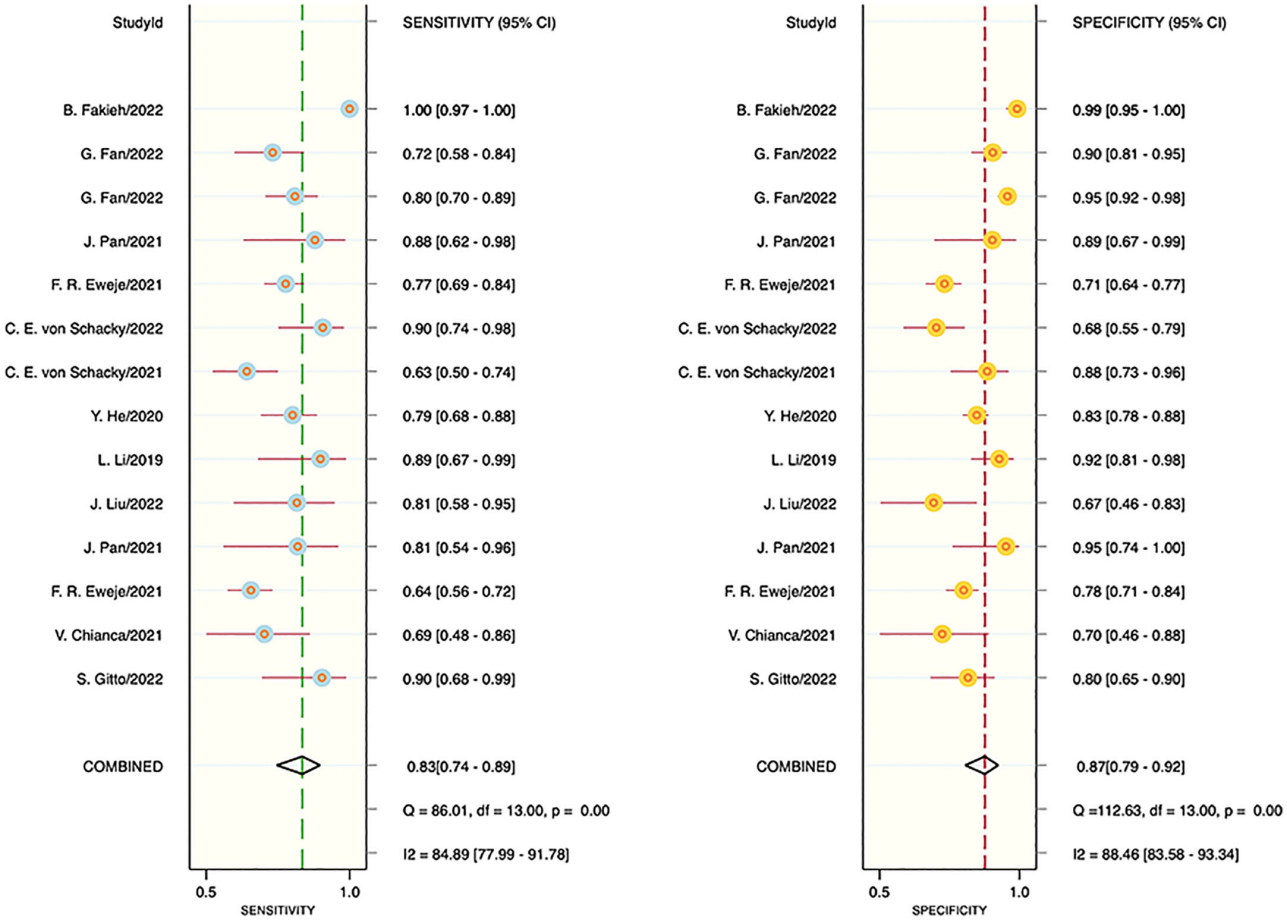
Forest plot for sensitivity and specificity in the validation set.

**Figure 5:**
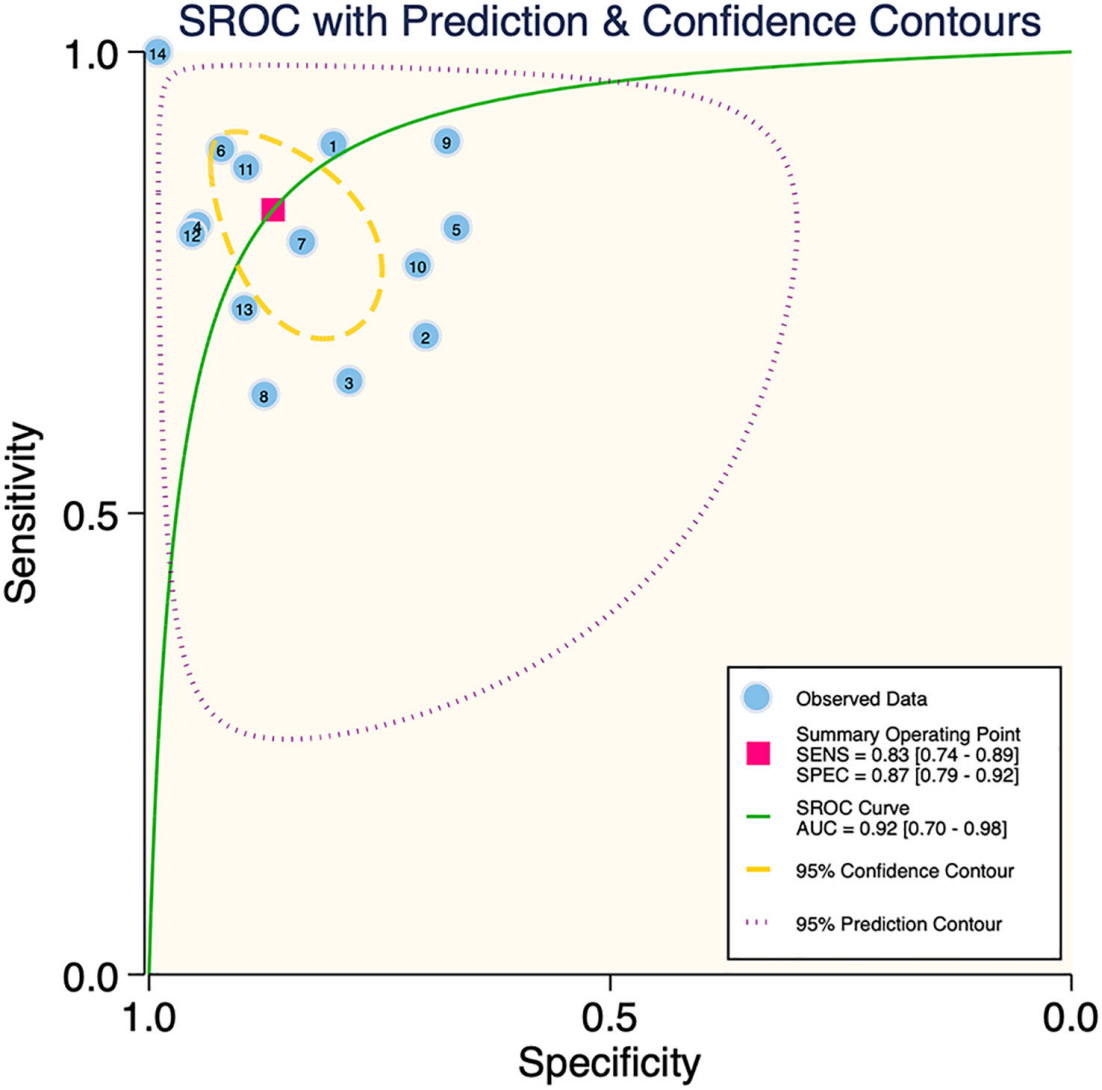
SROC plot of the validation set.

The results of the meta-analysis showed that in the training set, the overall diagnostic sensitivity of machine learning for malignant bone tumors was 0.87 [95% CI: 0.81,0.91]; the specificity was 0.91 [95% CI: 0.86,0.94]; the PLR was 9.4 [95% CI: 6.1,14.4]; the NLR was 0.14 [95% CI: 0.10, 0.21]; the DOR was 65 [95% CI: 33,127], and the SROC was 0.95 [95% CI: 0.19-1.00]. The forest plot for the sensitivity and specificity in the training set is shown in **Figure 2**, and the SROC curve of the training set is depicted in **Figure 3**. In the validation set, the overall diagnostic sensitivity of machine learning for malignant bone tumors was 0.83 [95% CI: 0.74,0.89]; the specificity was 0.87 [95% CI: 0.79,0.92]; the PLR was 6.2 [95% CI: 3.6,10.5]; the NLR was 0.20 [95% CI: 0.12,0.33]; the DOR was 31 [95% CI: 12,81], and SROC was 0.92 [95% CI: 0.70-0.98]. The forest plot for sensitivity and specificity in the validation set is shown in **Figure 4**, and the SROC plot of the validation set is illustrated in **Figure 5**.

### Publication bias and clinical applicability

**Figure 6:**
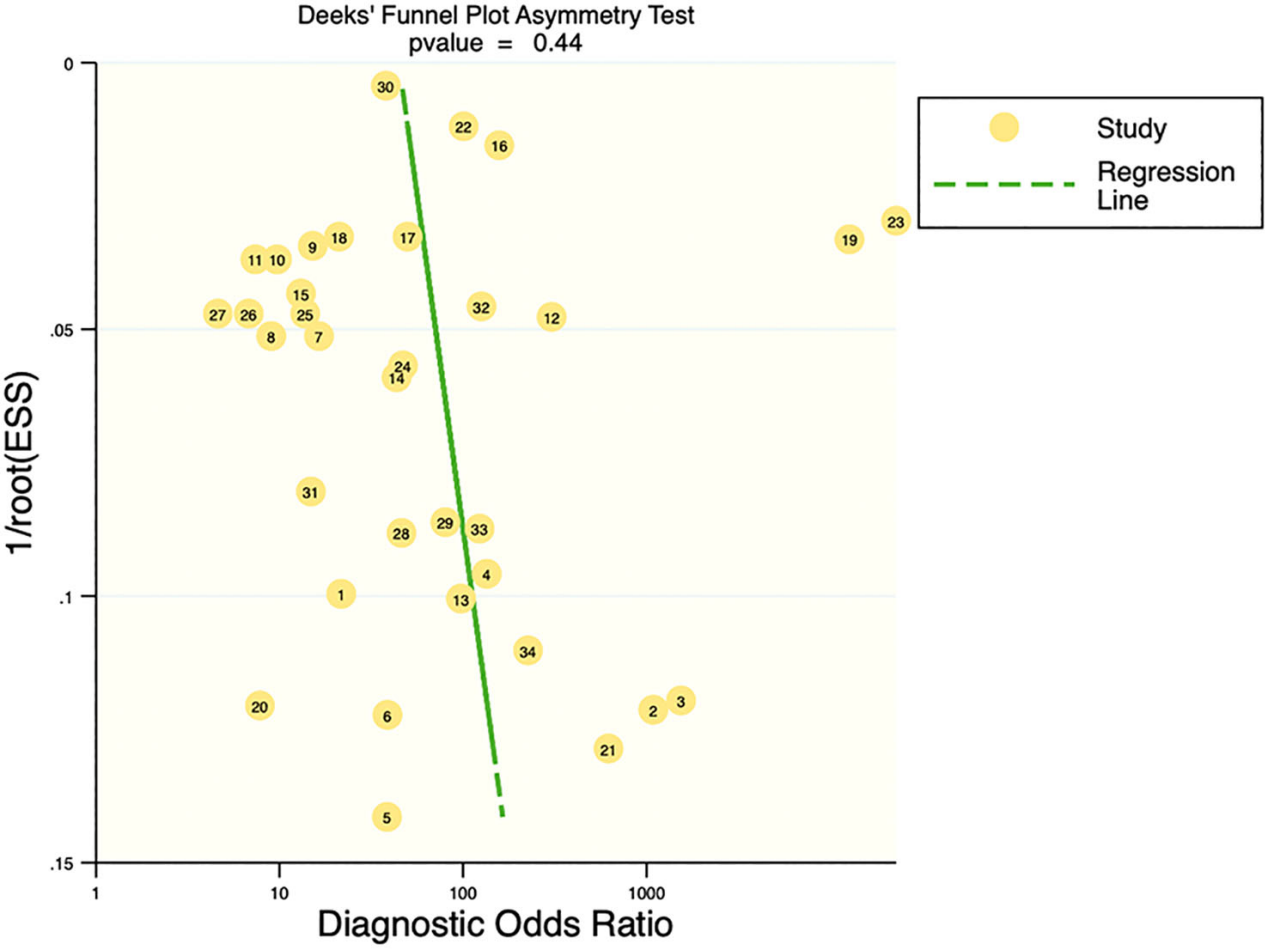
Deek’s plot of the training set.

**Figure 7:**
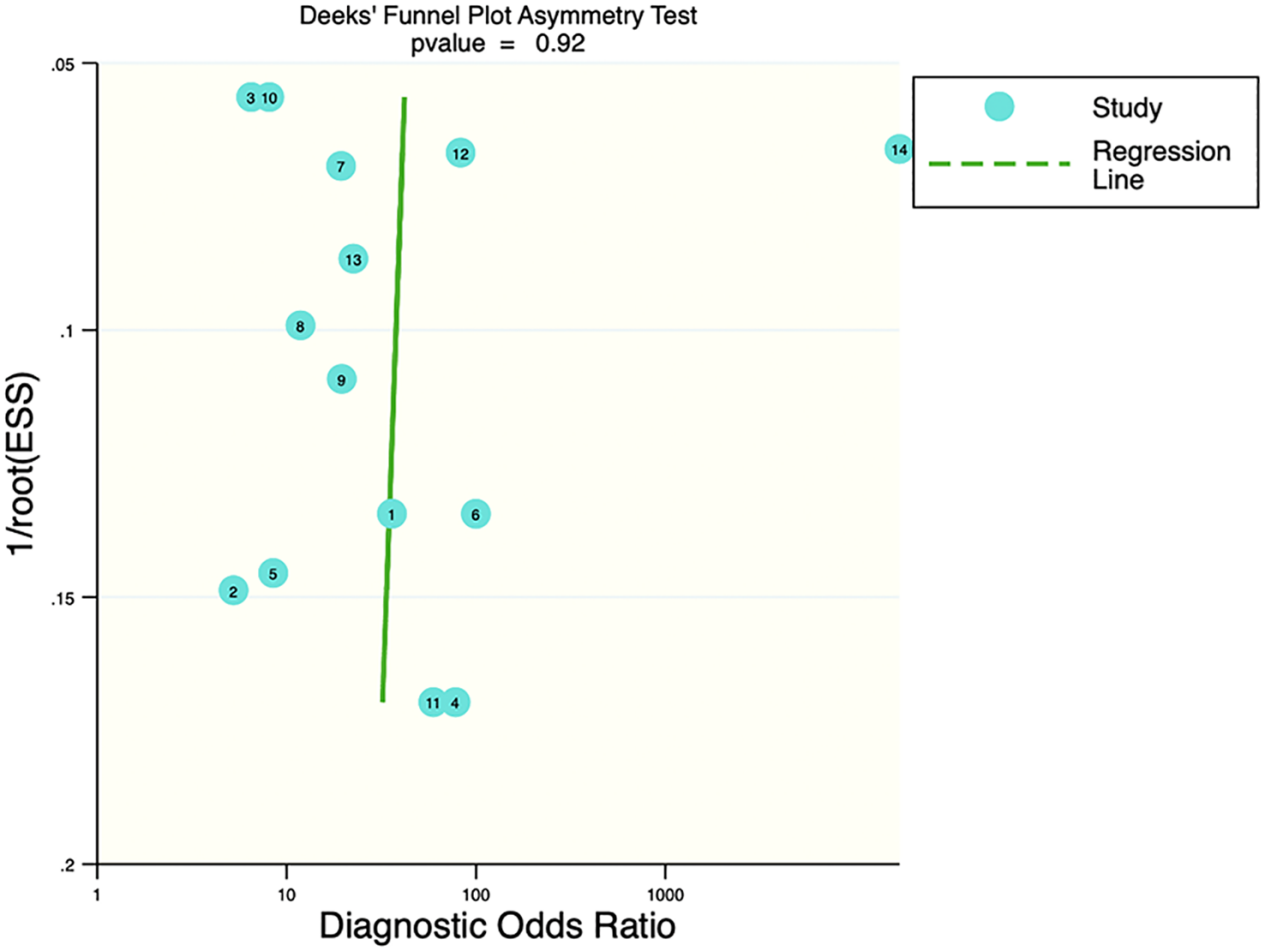
Deek’s plot of the validation set.

**Figure 8:**
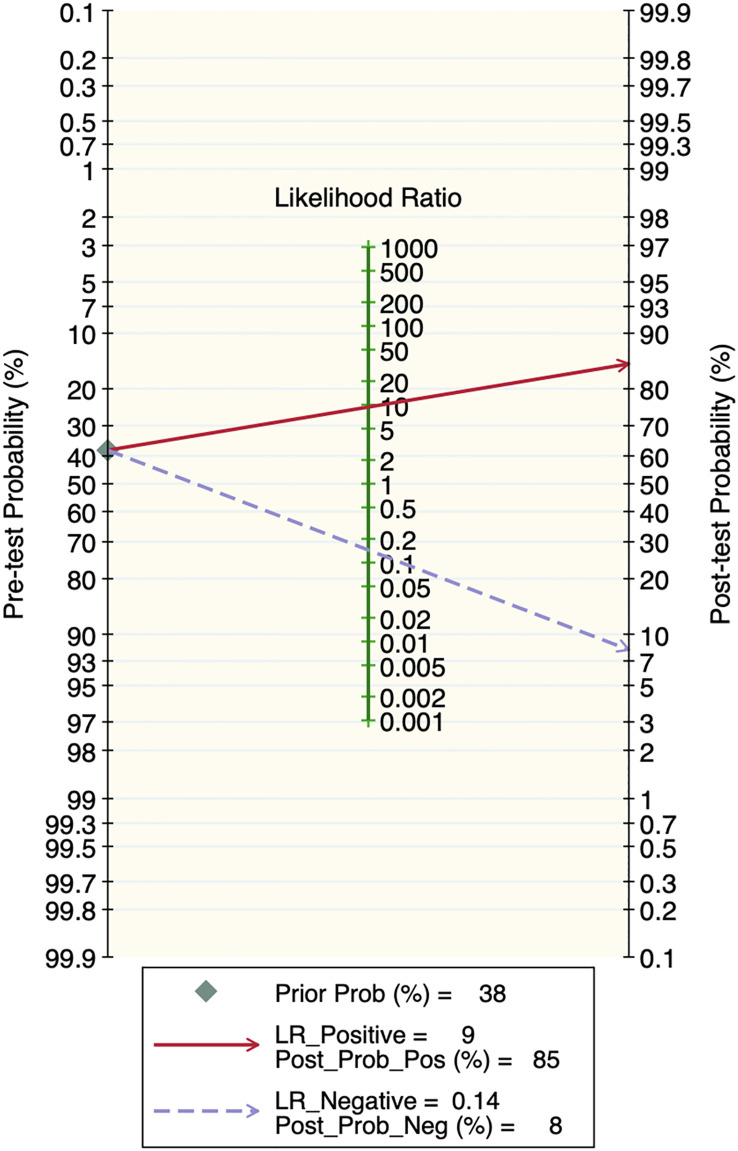
Clinical applicability plot of the training set.

**Figure 9:**
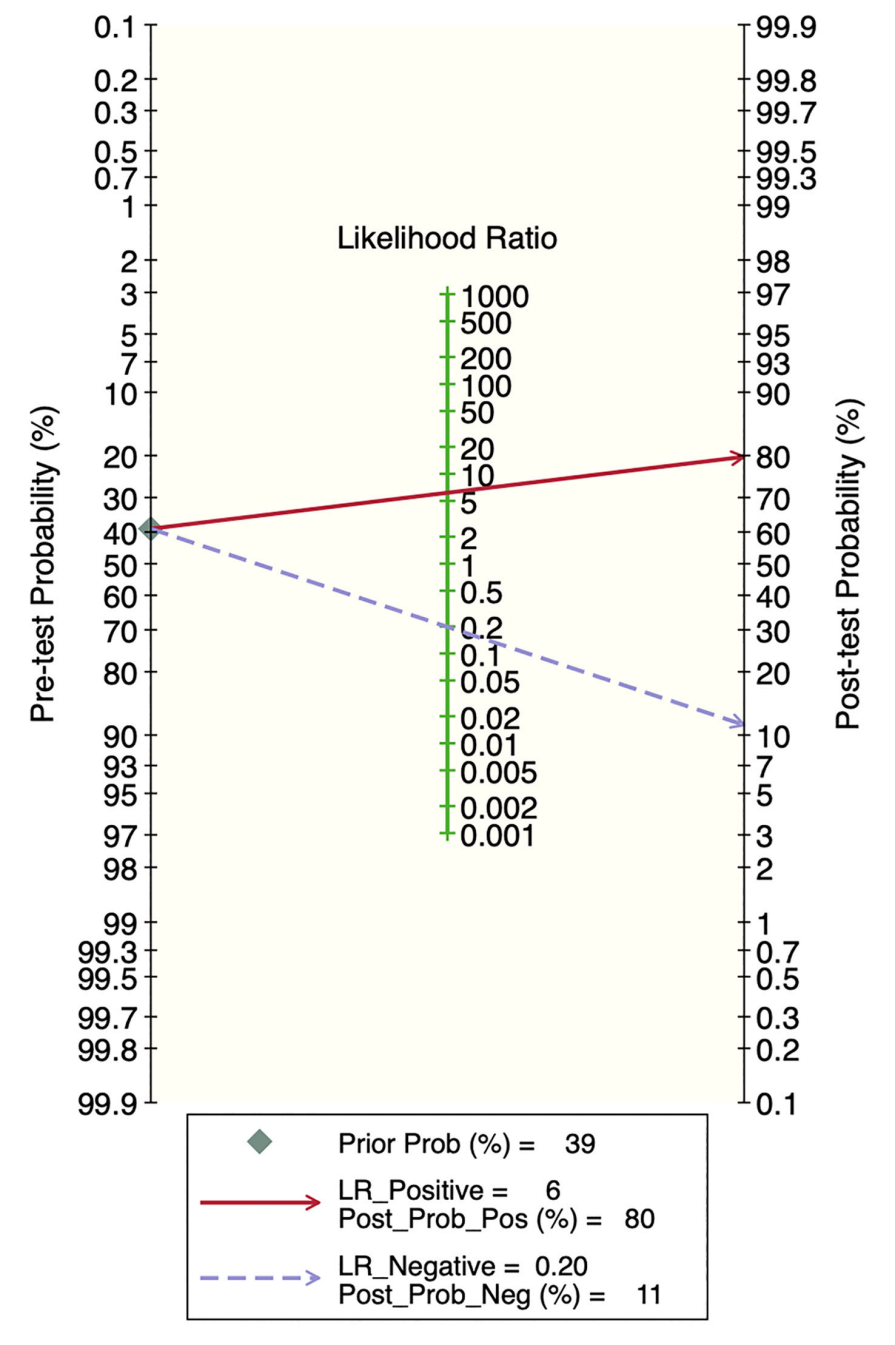
Clinical applicability plot of the validation set.

Deek’s funnel plot showed that there was no significant publication bias in both the training and validation sets (training set: P=0.44; validation set: P=0.92). The included studies showed that the prevalence of malignant bone tumors in the training set was approximately 38%. Therefore, the prior probability of the training set was assumed to be 38% when the clinical applicability was analyzed using nomograms. If machine learning diagnosed the lesion as a malignant bone tumor, the probability of it actually being a malignant bone tumor would be 85% (i.e., post-test probability=85%). If machine learning diagnosed the lesion as a non-malignant bone tumor, the probability of it actually being a malignant bone tumor would be 8%. Likewise, the prevalence of malignant bone tumors in the validation set was approximately 39%. In the analysis of clinical applicability using nomograms, the prior probability of the validation set was 39%. If machine learning diagnosed the lesion as a malignant bone tumor, the probability of it actually being a malignant bone tumor was 80% (i.e., post-test probability=80%). If machine learning diagnosed the lesion as a non-malignant bone tumor, then the probability of it actually being a malignant bone tumor was 11%. The publication bias and clinical applicability are shown in **Figures 6**–**9**.

### Subgroup analysis

According to the subgroup analysis by model types in the training set, the number of CNN-related literature was 13, accounting for the largest share. The overall diagnostic sensitivity was 0.86 [95% CI: 0.72,0.94]; specificity was 0.92 [95% CI: 0.82,0.97]; PLR was 11.3 [95% CI: 4.6,27.9]; NLR was 0.15 [95% CI: 0.07,0.32]; DOR was 74 [95% CI: 20,277], and SROC was 0.95 [95% CI: 0.62-1.00]. In the subgroup analysis by modeling variables in the training set, the number of MRI-related literature was 14, accounting for the largest share. The overall diagnostic sensitivity was 0.85 [95% CI: 0.74,0.91]; specificity was 0.87 [95% CI: 0.81,0.91]; PLR was 6.3 [95% CI: 4.1,9.6]; NLR was 0.18 [95% CI: 0.10,0.31]; DOR was 36 [95% CI: 14,87], and SROC was 0.92 [95% CI: 0.74-0.98].

According to the subgroup analysis by model types in the validation set, the number of CNN-related literature was 5, which accounted for the largest share. The overall diagnostic sensitivity was 0.87 [95% CI: 0.51,0.98]; specificity was 0.87 [95% CI: 0.69,0.96]; PLR was 6.9 [95% CI: 2.0,23.7]; NLR was 0.15 [95% CI: 0.03,0.84]; DOR was 46 [95% CI: 3,837], and SROC was 0.93 [95% CI: 0.59-0.99]. In the subgroup analysis by the modeling variables in the validation set, the number of MRI-related literature was 8, which accounted for the largest share. The overall sensitivity was 0.79 [95% CI: 0.70,0.86]; specificity was 0.79 [95% CI: 0.70,0.86]; PLR was 3.8 [95% CI: 2.5,5.7]; NLR was 0.26 [95% CI: 0.18,0.40]; DOR was 14 [95% CI: 7,30], and SROC was 0.86 [95% CI: 0.67-0.95]. The results of the subgroup analysis are shown in **Table 1**.

**Table 1:** Results of meta-analysis.

Training set results										
subgroup	Levels	Number of patients with malignant bone tumors	Number of negative events	Number	sen	spe	PLR	NLR	DOR	SROC
Model type	DT	1760	2658	2	0.89-0.95	0.88-0.98	8.19-66.21	0.05-0.10	157.47-620.50	
	LR	331	439	4	0.91[0.82,0.96]	0.97[0.91,0.99]	28.1[9.9,79.7]	0.09[0.04,0.20]	313[62,1587]	0.99[0.43-1.00]
	SVM	265	234	5	0.89[0.81,0.93]	0.84[0.74,0.90]	5.4[3.2,9.0]	0.14[0.08,0.24]	39[14,108]	0.93[0.18-1.00]
	XGBoost	252	776	2	0.62-0.69	0.84-0.87	3.98-5.64	0.34-0.44	9.01-16.40	
	RF	1460	1663	4	0.85[0.62,0.95]	0.86[0.73,0.94]	6.2[2.9,13.2]	0.18[0.06,0.50]	35[8,153]	0.92[0.62-0.99]
	CNN	138899	232164	13	0.86[0.72,0.94]	0.92[0.82,0.97]	11.3[4.6,27.9]	0.15[0.07,0.32]	74[20,277]	0.95[0.62-1.00]
	ANN	448	1022	4	0.84[0.69,0.93]	0.86[0.78,0.91]	6.0[3.3,10.7]	0.18[0.08,0.41]	33[8,128]	0.91[0.73-0.98]
Modeling variables	(18)F-FDGPET/CT	59	44	1	0.86	0.77	3.8	0.17	21.675	
	CT	2973	217313	5	0.82[0.59,0.93]	0.91[0.87,0.94]	9.0[5.6,14.4]	0.20[0.08,0.51]	45[12,165]	0.93[0.91-0.95]
	MRI	1913	2592	14	0.85[0.74,0.91]	0.87[0.81,0.91]	6.3[4.1,9.6]	0.18[0.10,0.31]	36[14,87]	0.92[0.74-0.98]
	X-Ray	1041	2906	7	0.80[0.68,0.89]	0.89[0.84,0.92]	7.1[4.7,10.9]	0.22[0.13,0.39]	32[13,79]	0.92[0.58-0.99]
	Pathological images	135430	16079	4	0.97[0.91,0.99]	0.97[0.73,1.00]	28.5[2.9,284.2]	0.03[0.01,0.10]	895[41,19374]	0.99[0.74-1.00]
	Clinical features	2069	3403	3	0.82[0.59,0.93]	0.91[0.87,0.94]	9.0[5.6,14.4]	0.20[0.08,0.51]	45[12,165]	0.93[0.91-0.95]
	LIBS	75	55	1	0.93	0.92	12.83	0.07	178.5	
Overall		143415	382371	34	0.87[0.81,0.91]	0.91[0.86,0.94]	9.4[6.1,14.4]	0.14[0.10,0.21]	65[33,127]	0.95[0.19-1.00]

Validation set results										
subgroup	Levels	Number of patients with malignant bone tumors	Number of cases	Number	sen	spe	PLR	NLR	DOR	SROC
Model type	DT	20	45	1	0.9	0.8	4.5	0.125	36	
	LR	37	46	2	0.80-0.81	0.66-0.94	2.42-15.43	0.19-0.28	8.50-78	
	SVM	19	51	1	0.89	0.92	11.4	0.11	99.87	
	AdaBoost	130	301	2	0.72-0.80	0.89-0.95	6.98-17.17	0.20-0.30	22.53-82.96	
	CNN	546	719	5	0.87[0.51,0.98]	0.87[0.69,0.96]	6.9[2.0,23.7]	0.15[0.03,0.84]	46[3,837]	0.93[0.59-0.99]
	ANN	31	65	1	0.9	0.67	2.79	0.14	19.55	
Modeling variables	CT	20	45	1	0.9	0.8	4.5	0.125	36	
	MRI	415	551	8	0.79[0.70,0.86]	0.79[0.70,0.86]	3.8[2.5,5.7]	0.26[0.18,0.40]	14[7,30]	0.86[0.67-0.95]
	X-Ray	138	263	2	0.62-0.79	0.83-0.87	4.78-5.02	0.24-0.42	11.84-19.38	
	Pathological images	122	106	1	1	0.99	106	0	NA	
	Clinical features	130	301	2	0.72-0.80	0.89-0.95	6.98-17.17	0.20-0.30	22.53-82.96	
Overall		825	1266	14	0.83[0.74,0.89]	0.87[0.79,0.92]	6.2[3.6,10.5]	0.20[0.12,0.33]	31[12,81]	0.92[0.70-0.98]

## Discussion

This study analyzed the accuracy of machine learning in the diagnosis of malignant bone tumors using meta-analysis. A total of 31 papers were included, including 382,371 samples, of which 141,315 were malignant bone tumor samples. The SROC was 0.95 in the training set and 0.93 in the validation set. It can be seen that machine learning is a feasible technique for the diagnostic identification of malignant bone tumors and has a good performance in radiomics, pathological images, and clinical features.

This systematic review showed that the most frequently used modeling variable was MRI. The overall diagnostic sensitivity and specificity of MRI were 0.85 [95% CI: 0.74,0.91] and 0.87 [95% CI: 0.81,0.91] in the training set, and 0.79 [95% CI: 0.70,0.86] and 0.79 [95% CI: 0.70,0.86] in the validation set, respectively. The favorable performance of MRI may be related to the nature of MRI itself and the characteristics of machine learning techniques. MRI can provide higher-resolution images and better soft tissue contrast, which can help machine learning algorithms to more accurately differentiate tissue types and detect lesion areas, thus improving the accuracy and reliability of the diagnosis (48, 50, 51). Xu Q et al. (52) established an MRI-based machine learning model for the identification of benign and malignant tumors in the kidney. The AUC of T2WI, DWI, and combined DL-based models in the test cohort were 0.906, 0.846, and 0.925, respectively. Ni M et al. (53) extracted, differentiated, and detected oblique coronal (OCOR) and oblique sagittal (OSAG) MRI images of the hip joint using a CNN model. LeNet-5 was applied to diagnose and classify lip trauma with an accuracy of 0.94/0.94 (OCOR) and 0.92/0.91 (OSAG), respectively, which helps radiologists to diagnose and classify upper lip injuries.

The machine learning models included in this study are DT, LR, SVM, XGBoost, RF, CNN, and ANN.

DTs have the advantage of being easy to understand and interpret, being tolerant of missing values, and being capable of handling unordered features. Yan W et al. used routine blood and biochemical test records of 4187 patients to establish an early auxiliary diagnostic model for multiple myeloma through DT, which had the highest precision (92.9%), recall (90.0%), and F1 score (0.915) compared to other models (SVM, DNN, RF) (45). However, DTs may overfit the data and are sensitive to noise and outliers (54).

LR is suitable for binary classification problems, easy to implement, and can provide probabilities for each predicted category. Pan J et al. used LR to construct a clinical feature + radiomics nomogram, which showed good performance in distinguishing malignant chondrosarcomas from benign enchondromas. Among all patients, the performance of the clinical-radiomics chart based on T2WI-FS (AUC = 0.967) was superior to that based on T2WI-FS (AUC = 0.901, P < 0.05) (35). However, LR assumes that the data is linearly separable and may not perform well when dealing with complex nonlinear relationships (55).

SVMs perform excellently when dealing with high-dimensional data and small sample data and have good generalization ability (56). Gitto S et al. used machine learning to differentiate benign from malignant in MRI images of 101 histologically confirmed spinal bone tumor patients. The results showed that the SVM classifier, based on radiological features extracted from T2 images and ADC images, has a good application prospect in spinal bone tumor classification (26). However, SVM also has some drawbacks. Its training process may be slow, and it is difficult to interpret.

XGBoost can handle various types of data, has good predictive performance, and can prevent overfitting (57). Liu R et al. collected data of pathologically diagnosed bone tumors from 2012 to 2019. Using routine X-ray images of the lesions and potentially related clinical data, they used XGBoost to classify the tumors as benign or malignant, with an AUC of 0.827, which is better than the 0.819 of the participating radiologists (15). However, XGBoost may be more difficult to tune than some other models, and it may have problems when dealing with extremely unbalanced data sets (58).

RF has strong resistance to noise and outliers and can handle nonlinear and large-scale data. Pan D et al. included 796 patients with histologically confirmed bone tumors, and they built an RF model to classify tumors into benign and malignant based on conventional radiological features and potentially related clinical features, with an accuracy rate of 94.71% (34). However, it should be noted that RF may overfit, especially in the presence of significant noise, and may be somewhat deficient in interpretability (59).

ANN has strong predictive ability and can handle nonlinear and high-dimensional data. Chianca V et al. performed a retrospective analysis on patients with spinal lesions who underwent MRI examination using ANN. The best feature selection method-ML algorithm combination was selected by performing 10-fold cross-validation 10 times in the training data. For the 2-label classification, ML achieved 94% accuracy in the internal test queue, and 86% accuracy in the external queue using hCAD data (22). However, ANN may require a large amount of training data and computational resources, may overfit, and is usually difficult to interpret (60).

The limitations of CNN are similar to those of ANN (61). It was also found that CNN was the preferred machine learning model in the current radiomics research on the identification of malignant bone tumors. According to the meta-analysis, the overall sensitivity and specificity of CNN were 0.86 [95% CI: 0.72,0.94] and 0.92 [95% CI: 0.82,0.97] in the training set, and 0.87 [95% CI: 0.51,0.98] and 0.87 [95% CI: 0.69,0.96] in the validation set. The advantage of CNN is that it can automatically learn and extract features from the input data without the need to extract features manually, which enables CNN models to excel in image, speech, natural language processing, and other fields. Moreover, the convolutional layers in CNN models are locally connected and weight-sharing, which significantly reduces the number of parameters of a CNN model and improves its training speed and generalization ability (62, 63). Gao Y et al. (64) developed a deep convolutional neural network (dCNN) model. The model is capable of automatically evaluating ultrasound images and can diagnose ovarian cancer more accurately than existing methods. The AUC of the dCNN model was 0.911 [95% CI: 0.886-0.936] in the internal dataset and 0.870 (95% CI 0.822-0.918) in the external validation dataset. The diagnostic performance of CNN ultrasound exceeded the average diagnostic level of radiologists. Tang F (65) et al. developed a CNN-based machine learning system that uses images from three optical coherence tomography (OCT) devices to classify Diabetic macular edema (DME). AUCs of 0.937 [95% CI 0.920-0.954], 0.958 [95% CI: 0.930-0.977], and 0.965 [95% CI: 0.948-0.977] were achieved in the primary datasets obtained with CIRRUS, SPECTRALIS and Triton OCT, respectively. Therefore, based on these findings, it seems possible to develop CNN-based intelligent auxiliary diagnostic tools to help clinicians identify malignant bone tumors.

In this study, we can see the extensive applications and potential of machine learning in the medical field, particularly in the early differential diagnosis of malignant bone tumors. In fact, with the advancement of technology, machine learning has found broad applications in many other fields, including but not limited to computer vision, pattern recognition, and audio-visual processing. For example, some studies have employed machine learning to address the problems of crowd counting and localization in computer vision (66, 67). These studies utilized complex machine learning models, such as hybrid classical-quantum networks and audio-visual dual-stream frameworks, to process and analyze image and audio data for accomplishing specific tasks. Another study used a novel multilayer neural network that integrates diffusion and drift memristors for image preprocessing and pattern recognition (68).

There are also some limitations to this study. First, although this study incorporated such modeling variables as radiomics, pathological images, and clinical features, there is a lack of literature on genomics combined with machine learning for the diagnosis of malignant bone tumors, which may lead to one-sided findings. Alge O et al. (69) created an RF model using features extracted from RNA-seq and x-ray image data to classify a given tumor as benign or osteosarcoma, and the proposed method achieved an AUC of 0.7272 with a triple characteristic curve and an AUC of 0.9015 with leave-one-out cross-validation. Barenboim M et al. (70) developed a new classifier based on DNA methylation patterns using machine learning and gene expression methods, which can detect BRCANES in osteosarcoma samples with high accuracy. Although these studies reported the value of machine learning for the diagnosis of malignant bone tumors, they were not included in this study due to the small number of patients enrolled.

Second, the number of studies involving external validation is small. For those studies that lacked external corroboration, the generalizability of their machine learning algorithms was not adequately assessed, and their reported performance should be interpreted with caution. Third, due to the lack of sufficient detail, subgroup analyses by populations with available key factors of DR were not performed, which may affect the clinical applicability of diagnostic tools. Fourth, poor reporting of the characteristics of patients included in the study may cause bias. Fifth, most studies were validated with retrospective data. The performance of machine learning may be overestimated in realistic settings due to spectral bias, and it should be considered.

Furthermore, some of the included literature did not provide detailed information on the types of malignant bone tumors studied or the number of patients involved. While this did not affect our analysis based on the c-index, sensitivity, and specificity, it may have impacted the evaluation of sample size and study quality. Secondly, the aim of this study was to investigate the value of machine learning in the diagnosis of malignant bone tumors, not to delve into each type of tumor in detail, which might introduce bias. Non-malignant conditions such as fractures were also categorized as non-malignant tumors to ensure sample size, but this may hinder the results from accurately reflecting the model’s ability to distinguish between malignant bone tumors and specific non-malignant conditions. Additionally, most literature did not report detailed tumor locations, potentially overlooking their influence on diagnosis. Finally, this study only covered a portion of malignant bone tumor types, reflecting the current limitations of research trends and data availability, which might limit the generalizability of the results. Future research should address these issues to better understand the potential of machine learning in the diagnosis of malignant bone tumors.

Overall, the results of this study suggest that machine learning can be of significant value in the diagnosis and differentiation between benign and malignant bone tumors, especially in improving diagnostic accuracy. However, despite the great advances of machine learning in medical image analysis, its application in clinical practice still needs more exploration and validation.

## Conclusion

In conclusion, the results of this study indicate that machine learning can serve as an effective means for early diagnosis of malignant bone tumors, and it is worth promoting for wider application. However, its practical efficiency and clinical applicability still require further exploration. Technically, future studies could explore the use of more advanced machine learning models or develop new, more effective feature extraction methods to improve the accuracy of diagnosis. In-depth studies conducted for specific types of tumors, specific stages of disease, or specific populations will contribute to understanding and applying the potential of machine learning in specific application scenarios. Cross-disciplinary collaborations should be carried out in the future, such as with bioinformatics, data science, and medical imaging, to promote the application of machine learning in tumor diagnosis. Moreover, future studies should establish more comprehensive models that cover a wider range of malignant bone tumors, and compare these models with traditional diagnostic methods in larger, multicenter studies. Additionally, integrating different types of data, such as clinical, pathological, and radiological imaging data, could also enhance the accuracy of the models. Furthermore, the application of machine learning in personalized treatment planning and prognosis prediction is another worthwhile avenue to explore. Finally, as the application of machine learning becomes integrated into healthcare, careful consideration must be given to ethical, legal, and societal impacts.

### Registration and protocol

This meta-analysis was carried out in accordance with the Preferred Reporting Items of Systematic Reviews and Meta-Analyses (PRISMA) guidelines. This systematic review was registered with PROSPERO, registration number CRD42023387057. The review protocol can be find on PROSPERO (https://www.crd.york.ac.uk/prospero/), any interpretation and modification of this protocol can be viewed on this website, which has been disseminated. All analyses were based on previous published studies; thus no ethical approval and patient consent are required.

## Data availability statement

The original contributions presented in the study are included in the article/**Supplementary Material**. Further inquiries can be directed to the corresponding author.

## Author contributions

YL and BD were responsible for conception and design. BD provided administrative support. YL was responsible for provision of study materials and patients. YL, PY, and BD were responsible for collection and assembly of data and data analysis and interpretation. All authors contributed to the article and approved the submitted version.
