# Supplementary material for: The diagnostic value of machine learning for the classification of malignant bone tumor: a systematic evaluation and meta-analysis
Source: Front Oncol. 2023 Sep 7;13:1207175. doi: 10.3389/fonc.2023.1207175 (PMC10513372; doi:10.3389/fonc.2023.1207175)
Supplement: Supplementary file 1 [file DataSheet_1.pdf]

Supplementary Material 1 Search strategy

1. Pubmed

Search number	Query	Results
1	"Sarcoma, Ewing"[Mesh]	7,567
2	"Bone Neoplasms"[Mesh]	133,941
3	"Osteosarcoma"[Mesh]	31,359
4	"Diaphyseal medullary stenosis with malignant fibrous histiocytoma" [Supplementary Concept]	1
5	"Chondrosarcoma"[Mesh]	7,407
6	"Bone Neoplasms"[Title/Abstract] OR "Bone Neoplasm"[Title/Abstract] OR "Neoplasm, Bone"[Title/Abstract] OR "Neoplasms, Bone"[Title/Abstract] OR "Bone Cancer"[Title/Abstract] OR "Cancer of the Bone"[Title/Abstract] OR "Cancer of Bone"[Title/Abstract] OR "Bone Lesions"[Title/Abstract] OR "Osteoblastic Bone Metastases"[Title/Abstract] OR "Bone Tumor"[Title/Abstract] OR "Bone Tumors"[Title/Abstract] OR "bone neoplasms"[Title/Abstract] OR "bone tissue neoplasms"[Title/Abstract] OR "neoplasms, bone tissue"[Title/Abstract] OR "skeletal tumor"[Title/Abstract] OR "skeletal tumour"[Title/Abstract] OR "skeleton tumor"[Title/Abstract] OR "skeleton tumour"[Title/Abstract] OR "tumor, bone"[Title/Abstract] OR "tumor, skeleton"[Title/Abstract] OR "tumour, bone"[Title/Abstract] OR "tumour, skeleton"[Title/Abstract]	21,208
7	"Ewing sarcoma"[Title/Abstract] OR "Ewing’s sarcoma"[Title/Abstract] OR "bone sarcoma, ewing"[Title/Abstract] OR "Ewing neoplasm"[Title/Abstract] OR "Ewing tumor"[Title/Abstract] OR "Ewing tumour"[Title/Abstract] OR "Ewing's neoplasm"[Title/Abstract] OR "Ewing's sarcoma"[Title/Abstract] OR "Ewing's tumor"[Title/Abstract] OR "Ewing's tumour"[Title/Abstract] OR "Ewings sarcoma"[Title/Abstract] OR "Ewings tumor"[Title/Abstract] OR "Ewings tumour"[Title/Abstract] OR "sarcoma, Ewing"[Title/Abstract] OR "sarcoma, Ewing's"[Title/Abstract] OR "Sarcoma, Ewings"[Title/Abstract]	9,241
8	"Osteosarcoma"[Title/Abstract] OR "bone sarcoma"[Title/Abstract] OR "intracortical sarcoma"[Title/Abstract] OR "osteogenic sarcoma"[Title/Abstract] OR "osteoid sarcoma"[Title/Abstract] OR "osteolytic sarcoma"[Title/Abstract] OR "primary osteogenic sarcoma"[Title/Abstract] OR "sarcoma, bone"[Title/Abstract] OR "sarcoma, osteogenic"[Title/Abstract] OR "Osteosarcomas"[Title/Abstract] OR "Sarcoma, Osteogenic"[Title/Abstract] OR "Osteogenic Sarcomas"[Title/Abstract] OR "Sarcomas, Osteogenic"[Title/Abstract] OR "Osteogenic Sarcoma"[Title/Abstract]	30,989
9	"multiple myeloma"[Title/Abstract] OR "Kahler disease"[Title/Abstract] OR "morbus kahler"[Title/Abstract] OR "myelomatosis"[Title/Abstract] OR "Multiple Myelomas"[Title/Abstract] OR "Myelomas, Multiple"[Title/Abstract] OR "Myeloma, Plasma-Cell"[Title/Abstract] OR "Myeloma, Plasma Cell"[Title/Abstract] OR "Myelomas, Plasma-Cell"[Title/Abstract] OR "Plasma-Cell Myeloma"[Title/Abstract] OR "Plasma-Cell Myelomas"[Title/Abstract] OR "Myeloma-Multiple"[Title/Abstract] OR "Myeloma Multiple"[Title/Abstract] OR "Myeloma-Multiples"[Title/Abstract] OR "Kahler Disease"[Title/Abstract] OR "Disease, Kahler"[Title/Abstract] OR "Plasma Cell Myeloma"[Title/Abstract] OR "Cell Myeloma, Plasma"[Title/Abstract] OR "Cell Myelomas, Plasma"[Title/Abstract] OR "Myelomas, Plasma Cell"[Title/Abstract] OR "Plasma Cell Myelomas"[Title/Abstract] OR "Myeloma, Multiple"[Title/Abstract] OR "Myelomatoses"[Title/Abstract]	45,913
10	"Diaphyseal medullary stenosis with malignant fibrous histiocytoma"[Title/Abstract] OR "DMSMFH"[Title/Abstract] OR "Bone dysplasia with malignant fibrous histiocytoma"[Title/Abstract] OR "Bone dysplasia with medullary fibrosarcoma"[Title/Abstract] OR "Chondrosarcoma"[Title/Abstract] OR "Chondrosarcomas"[Title/Abstract]	8,763
11	#1 OR #2 OR #3 OR #4 OR #5 OR #6 OR #7 OR #8 OR #9 OR #10	212,532

12	"Machine Learning"[Mesh]	48,517
13	"machine learning"[Title/Abstract] OR "Transfer Learning"[Title/Abstract] OR "Deep learning"[Title/Abstract] OR "Learning, Transfer"[Title/Abstract] OR "Ensemble Learning"[Title/Abstract] OR "artificial intelligence"[Title/Abstract] OR "Prediction model"[Title/Abstract] OR "random forest"[Title/Abstract] OR "neural network"[Title/Abstract] OR "Support vector machine"[Title/Abstract] OR "SVM"[Title/Abstract] OR "Gradient Boosting Machine"[Title/Abstract] OR "GBM"[Title/Abstract] OR "Nomogram"[Title/Abstract] OR "XGboost"[Title/Abstract] OR "Adaboost"[Title/Abstract] OR "Decision tree"[Title/Abstract] OR "Risk Prediction"[Title/Abstract] OR "Risk-Prediction"[Title/Abstract] OR "Radiomics"[Title/Abstract] OR "radiomic"[Title/Abstract] OR "Radiogenomic"[Title/Abstract]	236,091
14	#12 OR #13	241,388
15	#11 AND #14	903

2. Cochrane

Search number	Query	Results
1	MeSH descriptor: [Bone Neoplasms] explode all trees	1306
2	MeSH descriptor: [Sarcoma, Ewing] explode all trees	106
3	MeSH descriptor: [Osteosarcoma] explode all trees	301
4	MeSH descriptor: [Multiple Myeloma] explode all trees	1781
5	MeSH descriptor: [Chondrosarcoma] explode all trees	23
6	('Bone Neoplasms' OR 'Bone Neoplasm' OR 'Neoplasm, Bone' OR 'Neoplasms, Bone' OR 'Bone Cancer' OR 'Cancer of the Bone' OR 'Cancer of Bone' OR 'Bone Lesions' OR 'Osteoblastic Bone Metastases' OR 'Bone Tumor' OR 'Bone Tumors' OR 'bone neoplasms' OR 'bone tissue neoplasms' OR 'neoplasms, bone tissue' OR 'skeletal tumor' OR 'skeletal tumour' OR 'skeleton tumor' OR 'skeleton tumour' OR 'tumor, bone' OR 'tumor, skeleton' OR 'tumour, bone' OR 'tumour, skeleton'):ti,ab	11357
7	('Ewing sarcoma' OR 'Ewing's sarcoma' OR 'bone sarcoma, ewing' OR 'Ewing neoplasm' OR 'Ewing tumor' OR 'Ewing tumour' OR 'Ewing's neoplasm' OR 'Ewing's sarcoma' OR 'Ewing's tumor' OR 'Ewing's tumour' OR 'Ewings sarcoma' OR 'Ewings tumor' OR 'Ewings tumour' OR 'sarcoma, Ewing' OR 'sarcoma, Ewing's' OR 'Sarcoma, Ewings'):ab,ti	320
8	('Osteosarcoma' OR 'bone sarcoma' OR 'intracortical sarcoma' OR 'osteogenic sarcoma' OR 'osteoid sarcoma' OR 'osteolytic sarcoma' OR 'primary osteogenic sarcoma' OR 'sarcoma, bone' OR 'sarcoma, osteogenic' OR 'Osteosarcomas' OR 'Sarcoma, Osteogenic' OR 'Osteogenic Sarcomas' OR 'Sarcomas, Osteogenic' OR 'Osteogenic Sarcoma'):ti,ab	742
9	('multiple myeloma' OR 'Kahler disease' OR 'morbus kahler' OR 'myelomatosis' OR 'Multiple Myelomas' OR 'Myelomas, Multiple' OR 'Myeloma, Plasma-Cell' OR 'Myeloma, Plasma Cell' OR 'Myelomas, Plasma-Cell' OR 'Plasma-Cell Myeloma' OR 'Plasma-Cell Myelomas' OR 'Myeloma-Multiple' OR 'Myeloma Multiple' OR 'Myeloma-Multiples' OR 'Kahler Disease' OR 'Disease, Kahler' OR 'Plasma Cell Myeloma' OR 'Cell Myeloma, Plasma' OR 'Cell Myelomas, Plasma' OR 'Myelomas, Plasma Cell' OR 'Plasma Cell Myelomas' OR 'Myeloma, Multiple' OR 'Myelomatoses'):ti,ab	5347
10	('Diaphyseal medullary stenosis with malignant fibrous histiocytoma' OR 'DMSMFH' OR 'Bone dysplasia with malignant fibrous histiocytoma' OR 'Bone dysplasia with medullary fibrosarcoma' OR 'Chondrosarcoma' OR 'Chondrosarcomas' OR ' Chondroblastic sarcoma' OR 'Chondromucosarcoma'):ti,ab	80

11	MeSH descriptor: [Machine Learning] explode all trees	254
12	('machine learning' OR 'Transfer Learning' OR 'Deep learning' OR 'Learning, Transfer' OR 'Ensemble Learning' OR 'artificial intelligence' OR 'Prediction model' OR 'random forest' OR 'neural network' OR 'Support vector machine' OR 'SVM' OR 'Gradient Boosting Machine' OR 'GBM' OR 'Nomogram' OR 'XGboost' OR 'Adaboost' OR 'Decision tree' OR 'Risk Prediction' OR 'Risk-Prediction' OR 'Radiomics' OR 'radiomic' OR 'Radiogenomic'):ti,ab	13959
13	#1 OR #2 OR #3 OR #4 OR #5 OR #6 OR #7 OR #8 OR #9 OR #10	17638
14	#11 OR #12	13974
15	#13 AND #14	180

3. Embase

Search number	Query	Results
1	'bone tumor'/exp	181254
2	'ewing sarcoma'/exp	17596
3	'chondrosarcoma'/exp	13580
4	'osteosarcoma'/exp	41975
5	'multiple myeloma'/exp	95779
6	'bone neoplasm':ti,ab OR 'neoplasm, bone':ti,ab OR 'neoplasms, bone':ti,ab OR 'bone cancer':ti,ab OR 'cancer of the bone':ti,ab OR 'cancer of bone':ti,ab OR 'bone lesions':ti,ab OR 'osteoblastic bone metastases':ti,ab OR 'bone tumor':ti,ab OR 'bone tumors':ti,ab OR 'bone neoplasms':ti,ab OR 'bone tissue neoplasms':ti,ab OR 'neoplasms, bone tissue':ti,ab OR 'skeletal tumor':ti,ab OR 'skeletal tumour':ti,ab OR 'skeleton tumor':ti,ab OR 'skeleton tumour':ti,ab OR 'tumor, bone':ti,ab OR 'tumor, skeleton':ti,ab OR 'tumour, bone':ti,ab OR 'tumour, skeleton':ti,ab	26755
7	'osteosarcoma':ti,ab OR 'bone sarcoma':ti,ab OR 'intracortical sarcoma':ti,ab OR 'osteoid sarcoma':ti,ab OR 'osteolytic sarcoma':ti,ab OR 'primary osteogenic sarcoma':ti,ab OR 'sarcoma, bone':ti,ab OR 'osteosarcomas':ti,ab OR 'sarcoma, osteogenic':ti,ab OR 'osteogenic sarcomas':ti,ab OR 'sarcomas, osteogenic':ti,ab OR 'osteogenic sarcoma':ti,ab	38123
8	'multiple myeloma':ti,ab OR 'morbus kahler':ti,ab OR 'myelomatosis':ti,ab OR 'multiple myelomas':ti,ab OR 'myelomas, multiple':ti,ab OR 'myeloma, plasma-cell':ti,ab OR 'myeloma, plasma cell':ti,ab OR 'myelomas, plasma-cell':ti,ab OR 'plasma-cell myeloma':ti,ab OR 'plasma-cell myelomas':ti,ab OR 'myeloma-multiple':ti,ab OR 'myeloma multiple':ti,ab OR 'myeloma-multiples':ti,ab OR 'kahler disease':ti,ab OR 'disease, kahler':ti,ab OR 'plasma cell myeloma':ti,ab OR 'cell myeloma, plasma':ti,ab OR 'cell myelomas, plasma':ti,ab OR 'myelomas, plasma cell':ti,ab OR 'plasma cell myelomas':ti,ab OR 'myeloma, multiple':ti,ab OR 'myelomatoses':ti,ab	77869
9	'diaphyseal medullary stenosis with malignant fibrous histiocytoma':ti,ab OR 'dmsmfh':ti,ab OR 'bone dysplasia with malignant fibrous histiocytoma':ti,ab OR 'bone dysplasia with medullary fibrosarcoma':ti,ab OR 'chondrosarcoma':ti,ab OR 'chondrosarcomas':ti,ab OR 'chondroblastic sarcoma':ti,ab OR 'chondromucosarcoma':ti,ab	10693
10	'bone sarcoma, ewing':ab,ti OR 'ewing neoplasm':ab,ti OR 'ewing tumor':ab,ti OR 'ewing tumour':ab,ti OR 'ewings neoplasm':ab,ti OR 'ewings sarcoma':ab,ti OR 'ewing sarcoma':ab,ti OR 'ewings tumor':ab,ti OR 'ewings tumour':ab,ti OR 'sarcoma, ewing':ab,ti OR 'sarcoma, ewings':ab,ti	7071
11	'machine learning'/exp	331809

12	'machine learning':ti,ab OR 'transfer learning':ti,ab OR 'deep learning':ti,ab OR 'learning, transfer':ti,ab OR 'ensemble learning':ti,ab OR 'artificial intelligence':ti,ab OR 'prediction model':ti,ab OR 'random forest':ti,ab OR 'neural network':ti,ab OR 'support vector machine':ti,ab OR 'svm':ti,ab OR 'gradient boosting machine':ti,ab OR 'gbm':ti,ab OR 'nomogram':ti,ab OR 'xgboost':ti,ab OR 'adaboost':ti,ab OR 'decision tree':ti,ab OR 'risk prediction':ti,ab OR 'risk-prediction':ti,ab OR 'radiomics':ti,ab OR 'radiomic':ti,ab OR 'radiogenomic':ti,ab	348681
13	#1 OR #2 OR #3 OR #4 OR #5 OR #6 OR #7 OR #8 OR #9 OR #10	305273
14	#11 OR #12	536600
15	#13 AND #14	3111

4. Web of science

Search number	Query	Results
1	Diaphyseal medullary stenosis with malignant fibrous histiocytoma OR DMSMFH OR Bone dysplasia with malignant fibrous histiocytoma OR Bone dysplasia with medullary fibrosarcoma OR Chondrosarcoma OR Chondrosarcomas OR Chondroblastic sarcoma OR Chondromucosarcoma)	8947
2	multiple myeloma OR Kahler disease OR morbus kahler OR myelomatosis OR Multiple Myelomas OR Myelomas, Multiple OR Myeloma, Plasma-Cell OR Myeloma, Plasma Cell OR Myelomas, Plasma-Cell OR Plasma-Cell Myeloma OR Plasma-Cell Myelomas OR Myeloma-Multiple OR Myeloma Multiple OR Myeloma-Multiples OR Kahler Disease OR Disease, Kahler OR Plasma Cell Myeloma OR Cell Myeloma, Plasma OR Cell Myelomas, Plasma OR Myelomas, Plasma Cell OR Plasma Cell Myelomas OR Myeloma, Multiple OR Myelomatoses)	71379
3	Osteosarcoma OR bone sarcoma OR intracortical sarcoma OR osteogenic sarcoma OR osteoid sarcoma OR osteolytic sarcoma OR primary osteogenic sarcoma OR sarcoma, bone OR sarcoma, osteogenic OR Osteosarcomas OR Sarcoma, Osteogenic OR Osteogenic Sarcomas OR Sarcomas, Osteogenic OR Osteogenic Sarcoma)	43834
4	Ewing sarcoma OR Ewing’s sarcoma OR bone sarcoma, ewing OR Ewing neoplasm OR Ewing tumor OR Ewing tumour OR Ewing's neoplasm OR Ewing's sarcoma OR Ewing's tumor OR Ewing's tumour OR Ewings sarcoma OR Ewings tumor OR Ewings tumour OR sarcoma, Ewing OR sarcoma, Ewing's OR Sarcoma, Ewings)	11926
5	Bone Neoplasms OR Bone Neoplasm OR Neoplasm, Bone OR Neoplasms, Bone OR Bone Cancer OR Cancer of the Bone OR Cancer of Bone OR Bone Lesions OR Osteoblastic Bone Metastases OR Bone Tumor OR Bone Tumors OR bone neoplasms OR bone tissue neoplasms OR neoplasms, bone tissue OR skeletal tumor OR skeletal tumour OR skeleton tumor OR skeleton tumour OR tumor, bone OR tumor, skeleton OR tumour, bone OR tumour, skeleton)	212977
6	#1 OR #2 OR #3 OR #4 OR #5	311773
7	machine learning OR Transfer Learning OR Deep learning OR Learning, Transfer OR Ensemble Learning OR artificial intelligence OR Prediction model OR random forest OR neural network OR Support vector machine OR SVM OR Gradient Boosting Machine OR GBM OR Nomogram OR XGboost OR Adaboost OR Decision tree OR Risk Prediction OR Risk-Prediction OR Radiomics OR radiomic OR Radiogenomic)	1943486
8	#6 AND #7	3892

Supplementary Material 2 Basic Information Sheet

No.	First author	Year	Author's nationality	Type of study	Patient source	Type of malignant bone tumor	Number of malignant bone tumor samples	Total number of samples	Number of malignant bone tumor samples in the training set	Total number of samples in the training set	Number of malignant bone tumor samples in the validation set	Number of samples in the validation set
1	R. Xu	2014	Japan	Retrospective	Single-center	Malignant bone tumor	59	103				
2	C. L. Xia*	2018	China	Retrospective	Single-center	Malignant bone tumor	334	600	267	480	69	120
3	J. Pan	2021	China	Retrospective	Single-center	Chondrosarcoma	53	103	37	68	16	35
4	L. Li	2019	China	Retrospective	Single-center	Chondrosarcoma	56	210	37	140	19	70
5	R. Kumar	2016	India		Single-center	Malignant bone tumor	50	100	25	50	25	50
6	J. Liu	2022	China	Retrospective	Single-center	Multiple myeloma	103	241	82	193	21	48
7	R. Liu	2022	China	Retrospective	Single-center	Malignant bone tumor	158	643	126	514	32	129
8	Y. He	2020	China	Retrospective	Multicenter	Malignant bone tumor	360	1356	292	1065	68	291
9	F. R. Eweje	2021	United States	Retrospective	Multicenter	Malignant bone tumor	478	1060	335	742	143	318
10	D. Pan	2021	China	Retrospective	Multicenter	Malignant bone tumor	215	796	151	557	64	189
11	A. Sharma	2021	India	Retrospective	Single-center	Malignant bone tumor	65	105	None	None	40	105
12	Y. Tao	2021	China	Case control	Single-center	Malignant bone tumor	156	458	109	320	47	138
13	C. E. von Schacky	2021	Germany	Retrospective	Multicenter	Malignant bone tumor	267	934	187	654	80 (internal) + 70 (external)	280 (internal) + 110 (external)
14	W. Yan	2021	China	Retrospective	Single-center	Multiple myeloma	1741	4325			348	837
15	P. Yin	2021	China	Retrospective	Single-center	Bone tumor	580	795			None	None
16	B. Fakieh	2022	Saudi Arabia		Database	Osteosarcoma	608	1144	486	915	122	228
17	S. Gitto	2022	Italy	Retrospective	Single-center	Malignant bone tumor of the spine	79	101				
18	S. Gitto	2022	Italy	Retrospective	Multicenter	Chondrosarcoma	39	158	19	93	20	65
19	C. Loraksa*	2022	Thailand	Retrospective	Single-center	Osteosarcoma	2212	269025	1769	215220	443	53085
20	A. Mosquera Orgueira	2022	Spain	Retrospective	Database	Multiple myeloma						

21	M. U. Nasir*	2022	Korea	Retrospective	Database	Osteosarcoma	608	1144				
22	C. W. Park	2022	Korea	Retrospective	Single-center	Malignant bone tumor	94	538				
23	C. E. von Schacky	2022	Germany	Retrospective	Multicenter	Malignant bone tumor	213	880	149	616	31	96
24	J. Wu	2022	China	Retrospective	Single-center	Osteosarcoma	204	204	164	164		40
25	J. Wu	2022	China	Retrospective	Multicenter	Osteosarcoma	198	198	168	168		30
26	M. C. F. Yeung*	2022	China	Retrospective	Database	Chondrosarcoma		149130		134227		14913
27	K. Zhao	2022	China	Retrospective	Single-center	Malignant bone tumor	212	304	125	180	87	124
28	S. Chen	2022	China	Case control	Single-center	Multiple myeloma	179	531				
29	X. Chen	2021	China	Case control	Single-center	Multiple myeloma	75	130				
30	V. Chianca	2021	Italy	Retrospective	Single-center	Malignant bone tumor of the spine	97	1146	71	100	26	46
31	G. Fan	2022	China	Retrospective	Multicenter	Multiple myeloma	382	1418	252	933	76 internal + 54 external	290 internal + 141 external

Note: The references marked with * in the table indicate those studies that have only provided the number of images used for machine learning modelling, but not the number of patients.

Supplementary Material 3 Table of model characteristics

No.	First author	Generation method of the validation set	Overfitting methods	Treatment of missing values	Variable screening/feature selection methods	Type of model used	Modeling variables
1	R. Xu		10-fold cross-validation	VOI-based threshold segmentation	Adoption of three texture parameters	SVM	PET/CT
2	C. L. Xia	5-fold cross-validation	5-fold cross-validation		Gray Level Co-Occurrence Matrix	SVM	X-Ray
3	J. Pan	Random splitting		Normalization of images by using Radcloud	Statistical tests + LASSO + clinical experience	LR	MRI+clinical features
4	L. Li	Splitting by acquisition time	10-fold cross-validation	Min-max normalization was performed for each radiomics feature	MRMR was used first, followed by RFE	SVM	MRI
5	R. Kumar	Random splitting	10-fold cross-validation	Active contour model (or snake model)	Gray Level Co-Occurrence Matrix	SVM	CT
6	J. Liu	Random splitting	5-fold cross-validation	Data were filtered by using (LLL, LLH, LHH, HHH, HHL, HLL, HLH, and LHL)	Conventional Feature Selection (CFS)	LR	MRI
7	R. Liu	Random splitting		Images were labelled by using LabelImg	For continuous clinical features, we used Bartlett's test to test for homogeneity of variances and Kruskal-Wallis rank sum test to test for differences between groups. For	XGBoost	X-ray + clinical features

					nominal clinical features, we used Pearson's chi-square test and Fisher's exact test		
8	Y. He	Random splitting + external validation	5-fold cross-validation		Clinical experience	CNN	X-Ray
9	F. R. Eweje	Random splitting + external validation	4-fold cross-validation	N4 bias correction and intensity normalization for each image were performed by using SimpleITK	Imaging and clinical characteristic models (using logistic regression) were combined by using a superimposed ensemble method	CNN	MRI+clinical features
10	D. Pan	Random splitting	6-fold cross-validation	Recursive Feature Elimination (RFE)	Conventional imaging features + clinical experience	RF	X-ray + clinical features
11	A. Sharma	5-fold cross-validation	5-fold cross-validation		Describe with texture descriptors	SVM	X-Ray
12	Y. Tao	Random splitting	Iterative training	Data were enhanced and pre-processed by random rotation, random horizontal flip and the normalization of original images		CNN	Pathological tissue images
13	C. E. von Schacky	Random splitting + external validation				CNN	X-Ray
14	W. Yan	Random splitting + external validation		Exclude patients from the pool when the number of missing values is greater than a specified threshold of 3, and populate a normal value if it is less than the threshold	Stepwise regression + clinical experience	DT	Clinical features
15	P. Yin	5-fold cross-validation	5-fold cross-validation	Median of a specific variance vector is used instead of outliers	Spearman correlation and GBDT	RF	CT+clinical features
16	B. Fakieh	Random splitting	WDO growth fitness function (FF)	GF based	Deep transfer learning based on SqueezNet model	CNN	Pathological tissue images
17	S. Gitto	10-fold cross-validation	10-fold cross-validation	Image denoising, bias field correction, image resampling	Stability and significance based + stepwise regression	SVM	MRI
18	S. Gitto	External validation	10-fold cross-validation	Image resampling, gray level normalization and discretization	lasso+RFE	DT	CT
19	C. Loraksa	Random splitting	BCE loss function		None, including radiomics variables only	CNN	CT
20	A. Mosquera Orgueira	10-fold cross-validation	10-fold cross-validation		Mclust algorithm	COX	Clinical features

21	M. U. Nasir		Data enhancement techniques		None, including pathology image variables only	DL	Pathological tissue images
22	C. W. Park	5-fold cross-validation	5-fold cross-validation	Min-max standardized technique	None, including radiomics variables only	CNN	X-Ray
23	C. E. von Schacky	Random splitting + external validation			Stepwise regression	ANN/RF	MRI+clinical features
24	J. Wu	Random splitting	Data enhancement techniques		None, including radiomics variables only	CNN	MRI
25	J. Wu	Random splitting			None, including radiomics variables only	CNN	MRI
26	M. C. F. Yeung	Random splitting			None, including image variables only	CNN	Pathological tissue images
27	K. Zhao				None, including radiomics variables only	CNN	MRI
28	S. Chen				Stepwise regression	LR	Clinical features
29	X. Chen	10-fold cross-validation	10-fold cross-validation		PCA	ANN	LIBS
30	V. Chianca	10-fold cross-validation + external validation	10-fold cross-validation		Statistical tests	ANN	MRI
31	G. Fan	10-fold cross-validation	10-fold cross-validation		WrapperSubsetEval; PCA; CorrelationAttributeEval; GainRatioAttributeEval; CfsSubsetEval	AdaBoost	Clinical features

Supplementary Material 4 Diagnostic fourfold table (training set)

No.	Author	Year	Data_set	Model	Radiomics source	tp	fp	fn	tn	sen	spe	FPR	FNR	PLR	NLR	DOR
1	R. Xu	2014	Training set	SVM	(18)F-FDG PET/CT	51	10	8	34	0.86	0.77	0.23	0.14	3.8	0.18	21.68
3	J. Pan	2021	Training set	LR	MRI	34	0	1	33	0.97	1	0	0.03	NA	0.03	NA
3	J. Pan	2021	Training set	LR	MRI	34	1	1	32	0.97	0.97	0.03	0.03	32.06	0.03	1088
4	L. Li	2019	Training set	SVM	MRI	34	8	3	95	0.92	0.92	0.08	0.08	11.83	0.09	134.58
5	R. Kumar	2016	Training set	SVM	CT	21	3	4	22	0.84	0.88	0.12	0.16	7	0.18	38.5
6	J. Liu	2022	Training set	LR	MRI	71	3	11	18	0.87	0.86	0.14	0.13	6.06	0.16	38.73
7	R. Liu	2022	Training set	XGBoost	X-Ray	79	61	47	327	0.63	0.84	0.16	0.37	3.99	0.44	9.01
7	R. Liu	2022	Training set	XGBoost	X-Ray	88	48	38	340	0.7	0.88	0.12	0.3	5.65	0.34	16.4
8	Y. He	2020	Training set	CNN	X-Ray	232	157	60	616	0.79	0.8	0.2	0.21	3.91	0.26	15.17
9	F. R. Eweje	2021	Training set	CNN	MRI	191	49	144	358	0.57	0.88	0.12	0.43	4.74	0.49	9.69
9	F. R. Eweje	2021	Training set	CNN	MRI	265	138	70	269	0.79	0.66	0.34	0.21	2.33	0.32	7.38
10	D. Pan	2021	Training set	RF	X-Ray	141	18	10	388	0.93	0.96	0.04	0.07	21.06	0.07	303.93
11	A. Sharma	2021	Training set	SVM	X-Ray	62	7	3	33	0.95	0.83	0.18	0.05	5.45	0.06	97.43
12	Y. Tao	2021	Training set	CNN	Pathological images	91	22	18	189	0.83	0.9	0.1	0.17	8.01	0.18	43.43

13	C. E. von Schacky	2021	Training set	CNN	X-Ray	110	46	77	421	0.59	0.9	0.1	0.41	5.97	0.46	13.07
14	W. Yan	2021	Training set	DT	Clinical features	1661	301	80	2283	0.95	0.88	0.12	0.05	8.19	0.05	157.48
15	P. Yin	2021	Training set	RF	CT	532	136	48	259	0.92	0.66	0.34	0.08	2.66	0.13	21.11
15	P. Yin	2021	Training set	RF	CT	516	55	64	340	0.89	0.86	0.14	0.11	6.39	0.13	49.84
16	B. Fakieh	2022	Training set	CNN	Pathological images	478	2	8	427	0.98	1	0	0.02	210.97	0.02	12756.63
17	S. Gitto	2022	Training set	SVM	MRI	62	7	17	15	0.78	0.68	0.32	0.22	2.47	0.32	7.82
18	S. Gitto	2022	Training set	DT	CT	17	1	2	73	0.89	0.99	0.01	0.11	66.21	0.11	620.5
19	C. Loraksa	2022	Training set	CNN	CT	448	716	1321	212735	0.25	1	0	0.75	75.5	0.75	100.76
21	M. U. Nasir	2022	Training set	CNN	Pathological images	601	2	7	534	0.99	1	0	0.01	264.91	0.01	22923.86
22	C. W. Park	2022	Training set	CNN	X-Ray	77	39	17	405	0.82	0.91	0.09	0.18	9.33	0.2	47.04
23	C. E. von Schacky	2022	Training set	ANN	MRI	98	103	51	364	0.66	0.78	0.22	0.34	2.98	0.44	6.79
23	C. E. von Schacky	2022	Training set	ANN	MRI	112	84	37	383	0.75	0.82	0.18	0.25	4.18	0.3	13.8
23	C. E. von Schacky	2022	Training set	RF	MRI	61	61	88	406	0.41	0.87	0.13	0.59	3.13	0.68	4.61
24	J. Wu	2022	Training set	CNN	MRI	154	10	10	30	0.94	0.75	0.25	0.06	3.76	0.08	46.2
25	J. Wu	2022	Training set	CNN	MRI	159	6	9	34	0.95	0.85	0.15	0.05	6.31	0.06	100.11
26	M. C. F. Yeung	2022	Training set	CNN	Pathological images	130200	6853	4027	8050	0.97	0.54	0.46	0.03	2.11	0.06	37.98
27	K. Zhao	2022	Training set	CNN	MRI	80	6	45	50	0.64	0.89	0.11	0.36	5.97	0.4	14.81
28	S. Chen	2022	Training set	LR	Clinical features	154	15	25	337	0.86	0.96	0.04	0.14	20.19	0.15	138.39
29	X. Chen	2021	Training set	ANN	LIBS	70	4	5	51	0.93	0.93	0.07	0.07	12.83	0.07	178.5
30	V. Chianca	2021	Training set	ANN	MRI	67	2	4	27	0.94	0.93	0.07	0.06	13.68	0.06	226.13

Supplementary Material 5 Diagnostic fourfold table (validation set)

No.	Author	Year	Data_set	Model	Radiomics source	tp	fp	fn	tn	sen	spe	FPR	FNR	PLR	NLR	DOR
18	S. Gitto	2022	External validation set	DT	CT	18	9	2	36	0.9	0.8	0.2	0.1	4.5	0.13	36
30	V. Chianca	2021	External validation set	ANN	MRI	18	6	8	14	0.69	0.7	0.3	0.31	2.31	0.44	5.25
9	F. R. Eweje	2021	Validation set	CNN	MRI	92	38	51	137	0.64	0.78	0.22	0.36	2.96	0.46	6.5
3	J. Pan	2021	Validation set	LR	MRI	13	1	3	18	0.81	0.95	0.05	0.19	15.44	0.2	78
6	J. Liu	2022	Validation set	LR	MRI	17	9	4	18	0.81	0.67	0.33	0.19	2.43	0.29	8.5
4	L. Li	2019	Validation set	SVM	MRI	17	4	2	47	0.89	0.92	0.08	0.11	11.41	0.11	99.88
8	Y. He	2020	Validation set	CNN	X-Ray	54	37	14	186	0.79	0.83	0.17	0.21	4.79	0.25	19.39
13	C. E. von Schacky	2021	External validation set	CNN	X-Ray	44	5	26	35	0.63	0.88	0.13	0.37	5.03	0.42	11.85
23	C. E. von Schacky	2022	External validation set	ANN	MRI	28	21	3	44	0.9	0.68	0.32	0.1	2.8	0.14	19.56
9	F. R. Eweje	2021	Validation set	CNN	MRI	110	51	33	124	0.77	0.71	0.29	0.23	2.64	0.33	8.1
3	J. Pan	2021	Validation set	LR	MRI	14	2	2	17	0.88	0.89	0.11	0.13	8.31	0.14	59.5
31	G. Fan	2022	Validation set	AdaBoost	Clinical features	61	10	15	204	0.8	0.95	0.05	0.2	17.18	0.21	82.96
31	G. Fan	2022	External validation set	AdaBoost	Clinical features	39	9	15	78	0.72	0.9	0.1	0.28	6.98	0.31	22.53
16	B. Fakieh	2022	Validation set	CNN	Pathological images	122	1	0	105	1	0.99	0.01	0	106	0	NA
